# Gene discovery and differential expression analysis of humoral immune response elements in female *Culicoides sonorensis* (Diptera: Ceratopogonidae)

**DOI:** 10.1186/1756-3305-7-388

**Published:** 2014-08-21

**Authors:** Dana Nayduch, Matthew B Lee, Christopher A Saski

**Affiliations:** USDA-ARS, Center for Grain and Animal Health Research, Arthropod Borne Animal Diseases Research Unit, 1515 College Avenue, Manhattan, KS 66502 USA; Clemson University Genomics and Computational Biology Laboratory, Institute for Translational Genomics, BRC #310, 105 Collins St, Clemson, SC 29634 USA

**Keywords:** Innate immunity, Biting midge, Toll, Imd, JAK/STAT, RNAseq, Attacin, Cecropin, Defensin

## Abstract

**Background:**

Female *Culicoides sonorensis* midges (Diptera: Ceratopogonidae) are vectors of pathogens that impact livestock and wildlife in the United States. Little is known about their biology on a molecular-genetic level, including components of their immune system. Because the insect immune response is involved with important processes such as gut microbial homeostasis and vector competence, our aims were to identify components of the midge innate immune system and examine their expression profiles in response to diet across time.

**Methods:**

In our previous work, we *de novo* sequenced and analyzed the transcriptional landscape of female midges under several feeding states including teneral (unfed) and early and late time points after blood and sucrose. Here, those transcriptomes were further analyzed to identify insect innate immune orthologs, particularly humoral immune response elements. Additionally, we examined immune gene expression profiles in response to diet over time, on both a transcriptome-wide, whole-midge level and more specifically via qRTPCR analysis of antimicrobial peptide (AMP) expression in the alimentary canal.

**Results:**

We identified functional units comprising the immune deficiency (Imd), Toll and JAK/STAT pathways, including humoral factors, transmembrane receptors, signaling components, transcription factors/regulators and effectors such as AMPs. Feeding altered the expression of receptors, regulators, AMPs, prophenoloxidase and thioester-containing proteins, where blood had a greater effect than sucrose on the expression profiles of most innate immune components. qRTPCR of AMP genes showed that all five were significantly upregulated in the alimentary canal after blood feeding, possibly in response to proliferating populations of gut bacteria.

**Conclusions:**

Identification and functional insight of humoral/innate immune components in female *C. sonorensis* updates our knowledge of the molecular biology of this important vector. Because diet alone influenced the expression of immune pathway components, including their effectors, subsequent study of the role of innate immunity in biological processes such as gut homeostasis and life history are being pursued. Furthermore, since the humoral response is a key contributor in gut immunity, manipulating immune gene expression will help in uncovering genetic components of vector competence, including midgut barriers to infection. The results of such studies will serve as a platform for designing novel transmission-blocking strategies.

**Electronic supplementary material:**

The online version of this article (doi:10.1186/1756-3305-7-388) contains supplementary material, which is available to authorized users.

## Background

*Culicoides* biting midges (Diptera: Ceratopogonidae) are nuisance pests and some species are important vectors of disease-causing viruses, protists, and nematodes. In the US, *Culicoides sonorensis* transmits bluetongue virus and epizootic hemorrhagic disease virus to wild and domestic ruminants (e.g. sheep, deer, cattle), and has also shown potential to vector other viruses [1, 2]. While both sexes of midges feed on sugars in the form of extrafloral nectar, female *C. sonorensis* midges are anautogenous, requiring blood meals to initiate egg development. Since this process also serves as a means of pathogen acquisition from infected hosts, only female midges are disease vectors.

Arthropod vectors utilize physical and physiological defenses to combat microbes that may be present in the blood or sugar meal and to maintain homeostatic balance in gut bacterial populations. Physical defenses include the peritrophic matrix, which forms around the ingested blood meal and partitions microbes such as bacteria by size-exclusion [3]. A second line of defense involves the innate immune response, comprised of humoral and cellular components that act locally (e.g., epithelia, proximal to microbes) and/or systemically (i.e., fat body and hemolymph). Three major conserved signaling pathways that orchestrate the insect humoral immune response have been elucidated in model organisms such as fruit flies and mosquito vectors and include: Imd (Immune deficiency), Toll and JAK/STAT (Janus kinase/signal transduction and activators of transcription) [4]. In some dipteran flies, the Imd pathway is activated when peptidoglycan cell wall components of Gram-negative bacteria directly bind transmembrane peptidoglycan recognition protein (PGRP) receptors, pattern recognition receptors (PRRs) which are present on a variety of cells, especially barrier epithelia and fat body [4]. Imd activation results in the synthesis of antimicrobial peptides (AMPs) such as Diptericin via the Relish transcription factor [5]. The Toll pathway is activated by peptidoglycan components of Gram-positive bacterial cell walls and fungal glucans, and thus primarily responds to infections with these classes of microorganisms [4]. In the insect hemocoel, binding of these microbe-associated molecular patterns (MAMPs) to circulating PRRs triggers an extracellular serine protease cascade that eventually results in intracellular activation of NF-ƙB response elements and the transcription of Toll-induced AMPs. Alternatively, fungal proteolytic activity also activates the Toll pathway via the protease Persephone [6]. In the JAK/STAT pathway, three components, the Domeless receptor, the Janus Kinase Hopscotch, and the transcription factor STAT are at least partly involved in antiviral defenses in various flies [7, 8]. Relatively recently, more evidence is mounting that implicates both the Imd and Toll pathways in the dipteran antiviral defense repertoire as well, including defense against entomopathogenic viruses and arboviruses [9, 10].

AMPs are small, potent, antimicrobial effectors that are quickly synthesized by the insect fat body, hemocytes or epithelia in response to pathogen or microbe exposure [11, 12]. A majority are cationic at physiological pH, which facilitates interactions with microbial cell envelope components [13]. Immune studies in important insect vectors have demonstrated AMP upregulation in response to pathogen challenge either by natural or artificial routes. *Anopheles gambiae* presented with bacteria and malaria parasites upregulate *defensin* in the midgut and carcass and express this AMP in the salivary glands during late stages of infection [14–16]. Sandflies express AMPs in response to *Leishmania* infection and some AMPs, such as Attacin, are involved in anti-trypanosomal responses in tsetse flies [17–19]. AMPs and other effectors also participate in population control of non-pathogenic gut microbes. Larval dipterans are exposed to environmental bacteria through normal feeding activities and often harbor these indigenous microbiota transstadially [20–23]. Populations of gut-associated microbiota in adult insects are tightly regulated and reflect a balance between the immune response and bacterial tolerance [24–26]. In several vectors, a tripartite relationship between gut bacteria, pathogens, and the vector innate immune response has been demonstrated, including the impact such associations have on vector competence [27–29]. Thus, knowledge of the humoral response of blood feeding vectors helps not only in understanding their biology, but can also reveal mechanisms underlying refractoriness.

Innate immune responses in biting midges, including AMP expression, have not been investigated. In our previous work, we sequenced and annotated the transcriptome of adult female *C. sonorensis* and examined the responding transcriptome profiles of whole midges during various feeding states. In the current study, we identified and describe the components of the humoral immune response including receptors, signaling molecules and effectors from the Toll, Imd, and JAK/STAT pathways. Furthermore, we examined their differential activation on a transcriptome-wide level in whole female midges under different feeding states (teneral, blood and sucrose feeding over time). The gut-specific expression of selected AMPs in response to blood and sugar meals was quantified over time, and we found that blood feeding alone highly induced expression of five AMPs in the alimentary canal. This is the first description of these pathways in the midge, and likewise is the first look at temporospatial expression of AMP genes in relation to diet source. The role of these immune pathways in gut microbial ecology and vector competence in midges is discussed.

## Methods

### Humoral immune gene discovery

The adult female midge reference transcriptome has been previously described in [30]. In brief, female midges were unfed (teneral) or were exposed to different diets (blood or sucrose) and sampled at early (2, 6, 12 h post ingestion, pooled) or late (36 h post ingestion) time intervals. Total RNA from whole midges was used to prepare indexed temporospatial specific sequencing libraries and deep sequenced on an Illumia HiSeq2000. A *de novo* transcriptome was constructed and can be downloaded from the Transcriptome Shotgun Assembly deposited at DDBJ/EMBL/GenBank under the accession GAWM00000000 and bioproject 238338. The transcriptome is comprised of 19,041 unigene assemblies that can be found in the GenBank nucleotide database under the following accessions: GAWM01000001- GAWM01019041. Homology based annotation of the unigene set was carried out through comparisons to *Aedes aegypti* and *Culex quinquefasciatus* datasets, and the non-redundant protein database at GenBank. For the current study, functional signatures were determined by alignment to the Interpro (http://www.ebi.ac.uk/interpro) and ImmunoDB (cegg.unige.ch/insect/immunodb) databases to check for domains and orthologs, respectively, and to confirm correct annotation along with complete ortholog structure/function. Essentially, these methods were used to determine if the unigene deduced amino acid sequences contained complete domains and motifs associated with the immune components function and structure as defined in other arthropods.

### Transcriptome-wide expression profiles of humoral immune genes

Humoral immune genes were identified by searching the gene annotations and assigned GO terms, and by applying knowledge from other arthropod systems. Digital genome-wide gene expression profiles for female midges under different feeding and temporal conditions were described previously [30]. Briefly, treatment groups were comprised of: teneral (unfed, 2 d old), or those fed either 10% sucrose or blood and collected to represent early (2, 6, 12 h post-ingestion, pooled) or late (36 h post-ingestion) conditions; two biological replicates of each of these five treatment groups were collected and analyzed to determine condition-specific global gene expression profiles. Pairwise comparisons were made between and within diet source across time using the Tuxedo software package as we previously described [30], and statistically significant differences in gene expression were reported (P ≤ 0.01).

### Alignments of AMP genes

Multiple alignments of deduced peptide sequences were performed using CLC Genomics Workbench (http://www.clcbio.com). Insect sequences downloaded from NCBI were manually trimmed, inspected, and aligned with CLUSTALW.

### Antimicrobial peptide expression in *C. sonorensis*alimentary canal

*Culicoides sonorensis* midges (AK colony) were reared at the US Department of Agriculture Arthropod-Borne Animal Diseases Research Unit and maintained at 26°C, 70-80% relative humidity, with a 12–12 hour light–dark photoperiod. One to two day-old female adult midges were allowed to feed *ad libitum* for 1.5 h on a 10% sucrose solution or for 1 h on defribrinated sheep blood (Colorado Serum Company, Denver, CO) via an artificial membrane. Each feeding trial was replicated three times. At 3, 8, 12, and 24 h post feeding, midges (n = 15/time point per replicate) were anesthetized with carbon dioxide and removed for processing. The alimentary canal was dissected from each midge and pooled by time point for homogenization in Tri-Reagent (Ambion). Total RNA extraction was performed using a modified manufacturer’s protocol incorporating Bromo-3-chloro-propane in the extraction step and overnight ethanol precipitation. RNA quality was analyzed with a Nanodrop spectrophotometer and cDNA was synthesized from 500 ng total RNA using the QuantiTech Reverse Transcription kit following the manufacturer’s instructions (Qiagen, Valencia, CA). qRT-PCR detection was performed using a 5 PRIME RealMasterMix SYBR ROX kit (5 Prime, Gaithersburg, MD) according to the manufacturer’s protocol and run in 10 μl reactions consisting of primers diluted to a final concentration of 250 nM and cDNA templates diluted 1:10. To minimize variability, pipetting was performed using an Eppendorf epMotion 5070 platform and reactions run in triplicate on a Mastercycler ep realplex thermalcycler (Eppendorf, Hauppauge, NY) with the following parameters: 95°C for 2 min, followed by 40 cycles of 95°C for 15 s, 60°C for 20 s, 60°C for 15 s. Primer sequences are listed in Additional file 1, and include the reference gene *EF1b* [GenBank: GAWM01010754], which was previously identified as a candidate since it is not differentially-expressed across teneral or sucrose- or blood-fed midges [30]. C_T_ values were analyzed using the Relative Expression Software Tool [31], which allows for group wise comparison and statistical analysis of relative expression while accounting for differences in primer efficiencies.

## Results and discussion

### Components of the *C. sonorensis*humoral immune system in the transcriptome

The adult female transcriptome consists of 19,041 unigenes as described previously [30]. A search of the assigned Gene Ontology (GO terms) for humoral and immune returned 52 and 125 unigenes, respectively. However, searching of GO terms did not reveal all critical components of the pathways described below, and subsequent manual curation and searching using public resources revealed a total of 217 unigenes (~1.1% of the adult female midge) that make up or are involved in the insect humoral immune response. Three major conserved pathways in insect humoral immunity were revealed, including: Imd (Immune deficiency), Toll and JAK/STAT (Janus kinase/signal transduction and activators of transcription) with all or most components such as receptors, signaling intermediates, transcriptional regulators, effectors and regulators. All critical components were identified for Toll and JAK/STAT, but we did not identify two signaling components of the Imd pathway (Imd, FADD). Below we introduce and describe the detailed components of the midge humoral immune response.

### Imd pathway

The Imd pathway is part of the dipteran humoral antibacterial response that is activated when meso-diaminopimelic acid-containing peptidoglycan (DAP-PGN) binds transmembrane long-form peptidoglycan recognition proteins (PGRPs) [4, 32]. We confirmed the identity of seven long-form PGRPs in the midge transcriptome (Table 1). For immune signal transduction to ensue, activated PGRPs act through the adaptor Imd and subsequently FADD, which are two death-domain proteins that interact with DREDD (a Caspase-8 homolog). Interestingly, we did not identify orthologs for either Imd or FADD in the *C. sonorensis* transcriptome, although these have been identified in other nematocera [33], but a DREDD ortholog was identified [GenBank: GAWM01000519]. In *Drosophila*, DREDD cleaves the inhibitory domain from phosphorylated Relish, and the rel domain then translocates to the nucleus to induce expression of effectors such as antimicrobial peptides (AMPs) [32]. Relish is phosphorylated by a parallel component of the Imd pathway involving IAP (inhibitor of apoptosis), TAB2 (tak-associated binding protein), and several kinases, such as TAK1 (transforming growth factor activated kinase) and the IKK complex [4]. Orthologs for all components of this branching part of the Imd pathway were found in the transcriptome including: IAP2 [GenBank: GAWM01008211], TAB2 [GenBank:GAWM01006076], TAK1 [GenBank: GAWM01010356; GenBank: GAWM01012184], the ird5 ortholog IKK-beta [GenBank: GAWM01013537] and the key ortholog IKK-gamma, also known as Kenny [GenBank: GAWM01018250]. Two non-allelic sequences for TAK1 were identified (Table 1). This MAP3K also modulates the branch point between IMD and JNK (c-Jun N-terminal kinase) pathways, by phosphorylating both the IKK complex and JNKK (jun-kinase-kinase), respectively [34]. We also identified two Relish orthologs, with one [GenBank: GAWM01014885] likely being either *rel-2* (a *rel-1* paralog), or possibly a truncated isoform of *rel-1* [GenBank: GAWM01014884].

**Table 1:** **Components of the insect Immune Deficiency (Imd) pathway and antimicrobial peptides (AMPs) identified in the** ***C. sonorensis*** **transcriptome**

Description	Acc. No.	Seq. no.	***Aedes***Hit	***Culex***Hit	e-Value	Comments ^a^
**Receptors**						
Peptidoglycan Recognition Protein (Long; PGRP-LC)	GAWM01004359	m.21976	AAEL013112	CPIJ006560	4.49e-23	Complete; cytoplasmic, TM and PGRP domain
PGRP-LC	GAWM01003592	m.19794	AAEL014640	CPIJ006561	1.90e-40	Complete; cytoplasmic, TM and PGRP domain
PGRP-LC	GAWM01011033	m.42666	AAEL014640	CPIJ006561	1.90e-40	Complete; cytoplasmic, TM and PGRP domain
PGRP-LC	GAWM01011035	m.42672	AAEL014640	CPIJ006561	1.90e-40	Complete; cytoplasmic, TM and PGRP domain
PGRP-LC	GAWM01011037	m.42675	AAEL014640	CPIJ006561	7.24e-43	Complete; cytoplasmic, TM and PGRP domain
PGRP-LC	GAWM01011039	m.42683	AAEL014640	CPIJ006561	1.90e-40	Complete; cytoplasmic, TM and PGRP domain
PGRP-LC	GAWM01000194	m.10444	AAEL014989	CPIJ008514	1.30e-22	Complete; cytoplasmic, TM and PGRP domain
**Signaling**						
DREDD (Caspase-8)	GAWM01000519	m.11119	AAEL014148	CPIJ009056	4.93e-66	Complete; death related ced-3 nedd2-like; complete ICE domain
Inhibitor of apoptosis (IAP)	GAWM01008211	m.33483	AAEL006633	CPIJ019231	4.4e-146	Complete; 3 BIR and one ring domain
tak1-associated binding protein (TAB)	GAWM01006076	m.27286	n/a	CPIJ000820	5.27e-21	Confirmed; ubiquitin domain (CUE) present
tak1 (MAP3K)	GAWM01010356	m.40419	AAEL007035	CPIJ006370	3.82e-62	Complete; dual specificity kinase
tak1 (MAP3K)	GAWM01012184	m.47419	AAEL012659	CPIJ006370	8.20e-54	Complete; dual specificity kinase
I-Kappa-B Kinase 2 (IKK2, IKK-gamma), key/kenny	GAWM01018250	m.843	AAEL012510	CPIJ006917	2.02e-43	Complete; NEMO and UBAN motifs
I-Kappa-B Kinase 1 (IKK1, IKK-beta), ird5	GAWM01013537	m.5295	AAEL003245	CPIJ015672	0	Complete; kinase domain present
**Transcription**						
NF-kappaB transcription factor, Relish	GAWM01014884	m.58438	AAEL007624	CPIJ012236	5.09e-52	Complete; NF-kB/Relish; rel homology domain (RHD), IPT domain, ankyrin repeat domain, death-like domain.
NF-kappaB transcription factor, Relish	GAWM01014885	m.58439	AAEL007624	CPIJ012236	2.64e-27	Partial; possibly Rel-2 or truncated isoform; RHD only
**AMPs**						
Attacin-like AMP	GAWM01008443	m.3410	n/a	n/a	3.53e-07	Attacin-like AMP,one glycine-rich G domain (AA 56–115); no signal peptide
Attacin	GAWM01017969	m.7821	AAEL003389	n/a	1.39e-22	Complete; two glycine-rich domains (AA 75–191); signal peptide (AA 1–18)
Defensin	GAWM01019039	m.9997	n/a	n/a	1.46e-04	Complete and probable paralog; 6 cysteines present; signal AA 1-22
Defensin	GAWM01019040	m.9998	n/a	n/a	5.14e-08	Complete and probable paralog; 6 cysteines present; signal AA 1-21
Cecropin	GAWM01000005	m.10000	n/a	n/a	3.93e-14	Complete; cecropin family signature sequence AA 31–54; signal AA 1-23
**Regulators**						
Caudal homeobox protein	GAWM01004228	m.2158	AAEL014557	CPIJ802291	1.83e-82	Homeobox domain present
Poor imd response upon knock-in (PIRK); PIMS; RUDRA	GAWM01010231	m.4008	n/a	CPIJ014088	3.27e-09	Putative, needs functional confirmation
FAS-associated factor 1, caspar	GAWM01012793	m.49687	AAEL003579	CPIJ012219	0	Complete; has FAF1, UAS and UBS domains
Peptidoglycan Recognition Protein (Short form); PGRPSC2/SC3	GAWM01018647	m.9236	AAEL007039	CPIJ016770	4.37e-84	Complete; Short form PGRP domain, signal AA 1-16

^a^TM, transmembrane helix; AA, amino acids; BIR, baculovirus IAP repeat; NEMO, NF-kappaB essential modulator; UBAN, ubiquitin binding motif; AMP, antimicrobial peptide.

Regulation of the Imd pathway in insects includes both basal and inducible regulators that modulate the timing and amplitude of the immune response, respectively [35]. In *C. sonorensis,* we identified the inducible negative regulators PIRK (poor Imd response upon knock-in, also known as PIMS or RUDRA) [GenBank: GAWM01010231] and PGRP-SC2/SC3 (a short-form scavenger type of circulating PGRP) [GenBank: GAWM01018647] as well as the basal negative regulators Caspar (also known as FAS-associated factor 1, FAF1) [GenBank: GAWM01012793] and Caudal [GenBank: GAWM01004228].

### Toll pathway

Unlike the Imd pathway of humoral immune response, the Toll pathway functions solely in the systemic (e.g. fat body and hemolymph) recognition of microbes in insects. This is because microbial MAMPs (e.g. Lys-type peptidoglycan or fungal glucans) do not directly bind Toll receptors but instead are pre-processed by circulating PRRs including PGRP-SA and Gram-negative binding proteins 1 and 3 (GNBP1, GNBP3; also knows as Beta-1,3 Glucan Binding Proteins). These interactions start a protease cascade that eventually cleaves circulating pro-Spaetzle to the Toll-binding cytokine Spaetzle, after which signal transduction and effector expression ensues [36, 37]. The upstream humoral components of the Toll pathway that were identified in *C. sonorensis* include PGRP-SA [GenBank: GAWM01018051], three GNBP1 orthologs [GenBank: GAWM01002165; GenBank: GAWM01003712; GenBank: GAWM01004143] and GNBP3 [GenBank: GAWM01011997], three putative Spaetzle orthologs [GenBank: GAWM01001358; GenBank: GAWM01006049; GenBank: GAWM01012721], all without signal peptide, and one Spaetzle-1 ortholog, complete with signal sequence [GenBank: GAWM01015015] (Table 2).

**Table 2:** **Components of the insect Toll pathway identified in the** ***C. sonorensis*** **transcriptome**

Description	Acc. no.	Seq. no.	***Aedes***Hit	***Culex***Hit	e-Value	Comments ^a^
**Upstream signaling**						
Peptidoglycan Recognition Protein (Short); PGRP-SA	GAWM01018051	m.7996	AAEL009474	CPIJ007162	6.18e-17	Complete; PGRP amidase domain, signal AA 1-20
Gram-Negative Binding Protein (GNBP), or Beta-1,3-Glucan Binding Protein (BGBP); GNBP-1	GAWM01002165	m.15449	AAEL009176	CPIJ004321	3.81e-90	Complete; glycoside hydrolase, glucanase domains; signal AA 1-20
GNBP-1/BGBP-1	GAWM01003712	m.20067	AAEL009176	CPIJ004324	3.81e-90	Complete; glycoside hydrolase, glucanase domains; signal AA 1-29
GNBP-1/BGBP-1	GAWM01004143	m.21344	AAEL009176	CPIJ004321	3.81e-90	Complete; glycoside hydrolase, glucanase domains; signal AA 1-16
GNBP-3/BGBP-3	GAWM01011997	m.46772	AAEL000652	CPIJ013556	2.96e-38	Complete; GNBP domain, signal AA 1-25
Spaetzle-like cytokine, Spz3	GAWM01001358	m.13389	AAEL014950	CPIJ001752	3.56e-129	Putative, no signal
Spaetzle-like cytokine, Spz5	GAWM01006049	m.2718	AAEL001929	CPIJ009906	1.29e-44	Putative, no signal
Spaetzle-like cytokine, Spz6	GAWM01012721	m.49435	AAEL012164	CPIJ002281	2.42e-37	Truncated, no signal
Spaetzle-like cytokine, Spz1?	GAWM01015015	m.58907	AAEL000499	CPIJ014270	1.59e-35	Complete; either Spz1A or 1B; signal AA 1-31
**Receptors**						
Toll receptor	GAWM01015594	m.61585	AAEL009551	CPIJ013183	5.73e-106	Possible toll; LRR/TIR but no flanking CRRs
Toll receptor	GAWM01019001	m.9915	AAEL000633	CPIJ019764	0	Possible toll; LRR/TIR but no flanking CRRs
Toll receptor	GAWM01015706	m.62033	AAEL009551	CPIJ008497	4.24e-152	Complete toll (LRR, flanking CRR, TIR)
Toll receptor	GAWM01013057	m.50841	AAEL002583	CPIJ016598	0	Complete toll (LRR, flanking CRR, TIR)
Toll receptor	GAWM01013058	m.50847	AAEL002583	CPIJ016598	0	Complete toll (LRR, flanking CRR, TIR)
**Cell signaling**						
myeloid differentiation primary response protein 88 (MYD88)	GAWM01018790	m.948	AAEL007768	CPIJ018307	1.44e-46	Complete; death domain (DD), TIR domain
Ser/Thr Kinase, Pelle (IRAK1)	GAWM01001221	m.12898	AAEL006571	CPIJ015474	1.62e-92	Complete; N terminal DD and C terminal kinase
Ser/Thr Kinase, Pelle (IRAK1)	GAWM01011117	m.42913	AAEL006571	CPIJ015474	1.62e-92	Complete; N terminal DD and C terminal kinase
Tube (IRAK4)	GAWM01007838	m.32196	AAEL007642	CPIJ013746	1.93e-42	Complete; similar to other nematocera, *Cs*Tube has death domain and kinase domain with RD motif
cactus (IkappaB)	GAWM01009580	m.37494	AAEL001584	CPIJ004774	1.95e-16	Complete; ankyrin repeats AA 119-356
**Transcription**						
dorsal/dif (REL1)^b^	GAWM01010293	m.40244	AAEL014821	CPIJ801839	4.73e-95	Complete; confirmed to have N-terminal rel homology domain and C-terminal IPT domain
dorsal/dif (REL1)	GAWM01010294	m.40249	AAEL014821	CPIJ801839	4.73e-95	Complete; confirmed to have N-terminal rel homology domain and C-terminal IPT domain
dorsal/dif (REL1)	GAWM01010296	m.40254	AAEL014821	CPIJ801839	4.73e-95	Complete; confirmed to have N-terminal rel homology domain and C-terminal IPT domain
dorsal/dif (REL1)	GAWM01010297	m.40255	AAEL014821	CPIJ801839	4.73e-95	Complete; confirmed to have N-terminal rel homology domain and C-terminal IPT domain

^a^AA, amino acids; LRR, leucine rich region; CRR, cysteine rich region; TIR, Toll/interleukin-1 receptor domain; IPT, Ig-like, plexins, transcription factor domain.

^b^most likely only two of these unigene sequences are true paralogs, but due to the conserved RHD and IPT domains the assembly could not delineate during annotation.

All cell-associated components of the insect Toll pathway were identified in the *C. sonorensis* transcriptome. Insect Toll receptors have characteristic extracellular N-terminus leucine-rich repeats (LRR), at least two flanking cysteine-rich motifs (CRR) and intracellular Toll/IL-1 receptor (TIR) domains [38]. We identified two putative Toll receptors, which were complete except for CRR motifs: [GenBank: GAWM01015594; GenBank: GAWM01019001] and three complete Toll receptors [GenBank: GAWM01015706; GenBank: GAWM01013057; GenBank: GAWM01013058] (Table 2). Intracellular Toll signaling involves three death-domain containing proteins including the adaptor MyD88 and the mammalian IRAK1 and IRAK4 orthologs Pelle and Tube, respectively [4]. Complete orthologs for MyD88 [GenBank: GAWM01018790], Pelle [GenBank: GAWM01001221; GenBank: GAWM01011117] and Tube [GenBank: GAWM01007838] were found in the transcriptome. CsPelle and CsTube both contain typical death and kinase domains, and CsPelle has the GD dipeptide motif while CsTube has the RD dipeptide motif [39]. In *Drosophila* Tube, the kinase function has been evolutionarily lost; however, Tube proteins from the nematocerans *Aedes aegypti* [GenBank: AAEL007642], *Culex pipiens* [GenBank: CPIJ013746] and now *C. sonorensis* retain complete kinase domains [39, 40]. Transcription of effector molecules in the insect Toll pathway is controlled by the Rel-inhibitor, and IkappaB ortholog, Cactus, and the Rel-1 transcription factors Dorsal or Dif. In the *C. sonorensis* transcriptome, we identified a complete Cactus ortholog [GenBank: GAWM01009580], containing typical ankyrin repeats, as well as several Dorsal orthologs. The CsDorsal sequences represent at least two *dorsal* genes and possibly two additional spliceforms (Table 2), and the sequence was highly similar to that from the single-copy *dorsal* gene in mosquitoes (data not shown) [41].

### Antimicrobial peptides

**Figure 1:**
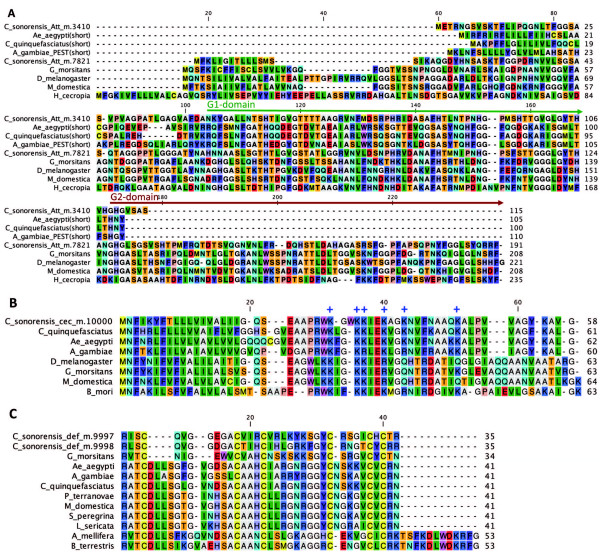
**ClustalW alignment of antimicrobial peptides from** ***Culicoides sonorensis*** **and other insects.** Deduced amino acid sequence for midge and other insect AMPs were compared and included: **(A)** Attacin family peptides, **(B)** Cecropins and **(C)** Defensins. Key features of each AMP are indicated in the alignment graphics: **(A)** Glycine-rich domains (G domains) of Attacins, **(B)** Conserved cationic amino acid residues (+) of Cecropins and **(C)** Six conserved cysteine residues (yellow) of Defensins. GenBank ID for protein sequences used in alignments were as follows: Attacins, *T. yao* ABX80077.1, *G. morsitans morsitans* CAP78961.1, *D. melanogaster* NP_523745.1, *C. capitata* XP_00451776.1, *A. gambiae* EAA11542.2, *C. quinquefasciatus* XP_001849658.1, *A. aegypti* EAT43734.1, *C. sonorensis* GAWM01008443 (attacin-like), *C. sonorensis* GAWM01017969 (attacin), *H. cecropia* AAA29183; Cecropins, *C. quinquefasciatus* XP_001861741.1, *A. aegypti* XP_001649178.1, *A. albimanus* ABS18287.1, *C.sonorensis* GAWM01000005*, G. morsitans morsitans* AAY41177.1, *D. melanogaster* AAF57026.1, *M. domestica* ABD38961.1, *B. mori* AAC60501.1; Defensins, *A. gambiae* ABB00948.1, *A. aegypti* XP_001657293.1, *C. pipiens pipiens* AAO38519.1*, L. sericata* ADI87383.1, *S. peregrina* P18313.1, *M. domestica* AAP33451.1, *P. terraenovae* P10891.2, *G. morsitans* Q8WTD4.1, *C. sonorensis* GAWM01019039 & GAWM01019040*, A. mellifera* AAA67443.1, *B. terrestris* ADB29129.1.

When Toll and Imd pathways are activated, their transcription factors (e.g. Dorsal, Relish) translocate to the nucleus and bind NF-kB promoters upstream of effector genes, such as those encoding antimicrobial peptides (AMPs). Full sequences for several AMPs were present in the midge transcriptome (Table 1). Two members of the Attacin superfamily were identified, with one having the full characteristics of insect Attacins [GenBank: GAWM01017969], bearing two C-terminus glycine-rich (G) domains in tandem (Figure 1A). The other attacin-like glycine-rich AMP [GenBank: GAWM01008443] had only one G domain, showing high similarity to the G1 domain of other dipteran glycine-rich AMPs (Figure 1A). This short CsAttacin-like AMP is not a Diptericin since it lacks both an N-terminus proline-rich P-domain and a pentaglycine repeat domain, which is characteristic of fly Diptericins [42, 43]. In mosquitoes, glycine-rich short AMPs annotated as “Attacins” also bear only one G domain (Figure 1A). Therefore, these nematoceran Attacin-like AMPs categorically are neither Diptericins nor Attacins, but rather represent another member of this AMP family. We infer that CsAttacin-like AMP is likely a truncated paralog of CsAttacin, rather than being an ortholog of the short Attacin-like AMPs in mosquitoes.

A single midge Cecropin was identified and is 58 amino acids in length including signal [GenBank: GAWM01000005] (Figure 1B). Cecropins have alpha-helical peptide structures that form pores in bacterial cell envelopes [11, 44, 45]. CsCecropin contains numerous, conserved positive amino acid residues (mainly lysine and arginine) which comprise a characteristic motif associated with this AMP class and is important in interactions with negatively charged bacterial cell membranes. The CsCecropin deduced amino acid sequence was most similar in sequence to Cecropins from other nematocera.

Two paralogous Defensins [GenBank: GAWM01019039; GenBank: GAWM01019040] were identified from the *C. sonorensis* transcriptome and only shared 34.8% sequence identity (Table 1, Figure 1C). Like other insect Defensins, both CsDefensins contain six conserved cysteines which are critical to the secondary structure of this AMP and the interaction with the bacterial envelope [46].

### JAK/STAT pathway

The JAK/STAT pathway is involved in the antiviral defense in insects, as well as cell proliferation, differentiation and development in flies such as *Drosophila* [10]. Viral infection causes upregulation of the cytokine Upd (unpaired), which is a ligand for the receptor Domeless (Dome). Pathway activation ensues after dimeric Dome receptors change conformationally and cause the autophosphorylation, and activation, of the JAK-kinase Hopscotch (Hop). Hop goes on to phosphorylate Dome, which provides STAT docking sites, after which Hop phosphorylates the SH2 domains on recruited STATs. Phosphorylated STAT dimers translocate to the nucleus and induce expression of target genes. We identified all components of the JAK/STAT pathway in the *C. sonorensis* transcriptome (Table 3) including partial [GenBank: GAWM01016058] and complete [GenBank: GAWM01016156] sequences for Dome, complete Hop [GenBank: GAWM01005626], and two partial [GenBank: GAWM01007780; GenBank: GAWM01011778] and one complete [GenBank: GAWM01013279] STAT. The mechanism by which the STAT-induced genes control viral amplification remains unknown, but reverse-genetic approaches have shown that *hop* mutant *Drosophila* have higher Drosophila-C virus (DCV) loads [7]. Similarly, RNAi knockdown of either *dome* or *hop* results in higher susceptibility to dengue virus infection in mosquitoes [8]. Two negative regulators of the JAK/STAT pathway include SOCS (suppressor of cytokine signaling), which prevents STAT activation by binding phosphorylated Hopscotch or by preventing or blocking docking sites on Dome receptors, and PIAS (protein inhibitor of activated STAT), which blocks STAT from accessing binding sites upstream of target genes [47]. Complete sequences for the JAK/STAT negative regulators SOCS and PIAS were identified. One of the *Culicoides* SOCS [GenBank: GAWM01008465] was structurally homologous to *Drosophila* SOCS36E, which has been confirmed to be a JAK/STAT repressor in flies [48], and the other [GenBank: GAWM01008657] is a possible ortholog of SOCS7. The two complete orthologs for the SUMO ligase PIAS [GenBank: GAWM01011450; GenBank: GAWM01011451] contain all the domains associated with the transcription-blocking functions of this inhibitor [49] (Table 3).

**Table 3:** **Components of the insect JAK/STAT pathway identified in the** ***C. sonorensis*** **transcriptome**

Description	Acc. no.	Seq. no.	***Aedes***hit	***Culex***hit	e-value	Comments ^a^
**Receptors**						
Domeless (Dome)	GAWM01016058	m.63662	AAEL012471	CPIJ017416	4.38e-85	Partial; Transmembrane Receptor, Domeless; has 2 fibronectin III (FNIII) domains
	GAWM01016156	m.64065	AAEL012471	CPIJ017416	4.38e-85	Complete; Transmembrane Receptor, Domeless; has 3 extracellular FNIII like domains
**Cell signaling**						
Hopscotch janus kinase (Hop)	GAWM01005626	m.25895	AAEL012553	CPIJ001760	0	Complete; Janus Kinase (JAK) signature; confirmed domains: ferm domain, SH2 domain, two TK domains
**Transcription**						
Signal transducer and activator of transcription (STAT)	GAWM01007780	m.32041	AAEL013265	CPIJ016471	1.01e-43	Partial; has p53 domain, SDE2 domain, STAT domain
	GAWM01011778	m.45760	AAEL013265	CPIJ016471	1.01e-43	Partial; missing STAT coiled coil domain
	GAWM01013279	m.51827	AAEL013265	CPIJ016471	1.01e-43	Complete; Stat-4 N-domain, AA 1–128; coiled coil, AA 132–327, STAT DNA-binding domain (P53 like), AA 328–467, EF-hand domain, AA 468–590, SH2 domain, AA 566-698
**Regulators**						
Suppressor of cytokine signaling 5 (SOCS36E?)	GAWM01008465	m.34186	AAEL000393	CPIJ003380	5.70e-102	Complete SOCS box, C-terminal; SH2 domain; confirmed in insects as JAK/STAT repressor
Suppressor of cytokine signaling (SOCS7?)	GAWM01008657	m.34752	AAEL006949	CPIJ003985	1.62e-104	Complete SOCS box, C-terminal; SH2 domain; homologous to Drosophila SOCS16D; function unknown
Protein inhibitor of activated stat; PIAS, sumo ligase	GAWM01011450	m.44071	AAEL015099	CPIJ009163	1.66e-150	Complete; SAP domain; PINIT domain; Zinc finger, MIZ-type; Zinc finger, RING/FYVE/PHD-type
	GAWM01011451	m.44074	AAEL015099	CPIJ009163	1.66e-150	Complete; SAP domain; PINIT domain; Zinc finger, MIZ-type; Zinc finger, RING/FYVE/PHD-type

^a^AA, amino acids; SH2, Src homology 2; TK, tyrosine kinase; SDE2, silencing defective 2.

### Other immune related genes

Other humoral immune components and effectors were found in the transcriptome including hemolymph defense molecules such as thioester-containing proteins (TEPs) and prophenoloxidase (PPO). Insect TEPs are active in the systemic response to invasive microbes, and help in opsonization for subsequent clearance by phagocytosis [50]. We identified two TEP3 orthologs [GenBank:GAWM01009528; GenBank: GAWM01016118] in *C. sonorensis*. In mosquitoes, TEP3 has been shown to be involved in both the antibacterial and antiparasitic (antimalarial) defense [50]. PPO zymogen is stored in insect hemocytes and is activated via a serine protease cascade to phenoloxidase (PO) in response microbial challenge. PO enzymes oxidize phenols to orthoquinones, which polymerize into melanin, and this results in melanization of invading microbes or wounds [4]. Two complete PPO paralogs were found in the *Culicoides* transcriptome [GenBank: GAWM01015170; GenBank: GAWM01010754]. Mosquitoes have from nine (*A. gambiae*) to ten (*Ae. aegypti*) genes coding for PPOs, and members of this family have been implicated in refractoriness to *Plasmodium* infection in *A. gambiae* [51].

### Dietary effects on transcriptome-wide expression of humoral immune genes

Many of the Imd, Toll and JAK/STAT genes were differentially expressed after female midges fed on blood or sucrose. The humoral immune response to diet is not due to direct stimulation, but rather is likely mediated through alteration of the gut microbial community. Such a circuitous influence on the gut epithelial immune response has been shown in mosquitoes, where diet causes proliferation of gut bacteria, which produce immunostimulatory MAMPs such as peptidoglycan (PGN). The diet promotes bacterial proliferation by two mechanisms: (1) directly, where the meal provides nutrients to support microbial growth or (2) indirectly, where components of the meal, such as free heme in blood, block the activity of reactive oxygen species that otherwise act in suppressing gut flora populations [52, 53]. The MAMPs produced by proliferating gut bacteria activate both local responses by binding PRRs on epithelial cells (e.g., Imd responses) and systemic responses in the hemocoel (e.g., Imd or Toll on the fat body), mediated through second messengers [54, 55] or by PGN diffusing into the hemolymph [56, 57]. A tripartite interaction between gut microbes, innate immune responses and vector competence for pathogens has been demonstrated in mosquitoes and other hematophagous insects [27, 29, 58–60]. In our gene expression analyses of the midge transcriptomes, we found that altered expression of humoral immune genes was more often associated with blood feeding than sugar feeding. Ongoing studies in our laboratory have shown that the blood meal induces proliferation of midge gut bacteria, and more specific analyses on these microbial-ecological dynamics are being assessed (data not shown).

**Figure 2:**
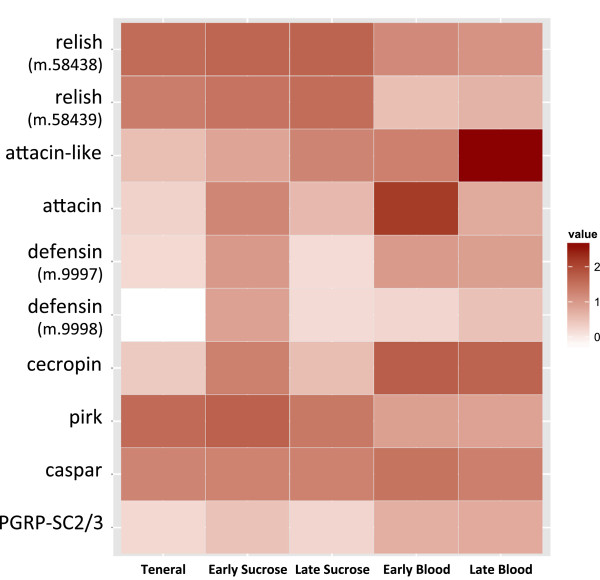
**Transcriptome-wide differential expression analyses of selected** ***Culicoides sonorensis*** **Imd and antimicrobial peptide genes.** Early transcriptomes are 2, 6, 12 h post ingestion (pooled) and late transcriptomes are 36 h post ingestion, for each diet (blood or sucrose). Teneral midges were newly emerged and unfed. Log_10_ FPKM values indicated in legend of the heat map. Further description of these genes can be found in Table 1, and fold-change values and statistics can be found in Table 4.

Expression of most of the Imd pathway genes changed significantly after female midges fed on blood or sucrose (P ≤ 0.01; Table 4). After feeding on blood or sucrose, three *PGRP* genes were upregulated, and three were down regulated, showing no clear pattern of response for these receptors. In regards to Imd cell signaling components, all were significantly upregulated after either early or late blood feeding (or both) except for *TAB2* and *TAK1*, which were not differentially expressed. *kenny* and *ird5* were downregulated in late blood fed midges relative to expression levels after early blood feeding (P ≤ 0.001; Table 4). Genes involved with feedback modulation of the Imd response were also differentially expressed in blood-fed midges. In early blood-fed midges, the negative regulators *pirk* and *caspar* were downregulated, which would permit early transcription of Imd-response target genes, including AMPs [35]. Expression of the transcription factor *relish* was downregulated in early blood fed-midges (Table 4, Figure 2), which may represent feedback mechanisms to modulate the amplitude of the immune response, which may represent feedback mechanisms to modulate the amplitude of the immune response. Transcripts coding for the scavenger amidase PGRP-SC2/SC3 were upregulated in late blood fed midges, possibly serving as a negative regulator to suppress excessive Imd stimulation [35].

**Table 4:** **Imd pathway and antimicrobial peptide (AMP) genes differentially expressed with diet**

Description	Acc. no.	Seq. no.	Diff. exp. ^a^	Log _2_fold chg	P-value
**Receptors**					
PGRP-LC	GAWM01003592	m.19794	Up (LS)	1.08	0.008
	GAWM01011033	m.42666	Up (EB to LB)	1.98	0.004
	GAWM01011035	m.42672	Up (EB to LB)	1.99	0.004
	GAWM01011037	m.42675	Down (EB)	-1.79	0.006
			Up (LB)	1.24	0.001
			Up (EB to LB)	3.04	1.61E-06
	GAWM01011039	m.42683	Down (EB)	-1.78	0.004
			Up (LB)	1.01	0.004
			Up (EB to LB)	3.04	2.95E-06
	GAWM01000194	m.10444	Down (EB)	-1.38	0.004
			Up (LB)	0.85	0.009
			Up (EB to LB)	2.23	2.05E-06
			Up (LS)	0.84	0.005
**Cell signaling**					
DREDD	GAWM01000519	m.11119	Up (EB)	1.71	2.85E-07
			Up (LB)	0.96	0.005
IAP2	GAWM01008211	m.33483	Up (EB)	1.24	0.0001
IKK-g/kenny	GAWM01018250	m.843	Up (EB)	1.82	5.18E-07
			Down (EB to LB)	-1.25	2.00E-04
IKK-b/ird5	GAWM01013537	m.5295	Up (EB)	1.54	8.19E-07
			Down (EB to LB)	-0.99	1.00E-03
**Transcription**					
Relish	GAWM01014884	m.58438	Down (EB)	-1.29	3.00E-04
			Up (EB to LB)	1.54	1.08E-05
	GAWM01014885	m.58439	Down (EB)	-2.79	2.00E-04
			Up (EB to LB)	3.03	5.23E-05
**AMPs**					
Attacin-like	GAWM01008443	m.3410	Up (EB)	2.63	1.00E-03
			Up (LB)	7.14	0
			Up (EB to LB)	4.5	2.89E-15
			Up (LS)	2.44	3.00E-04
Attacin	GAWM01017969	m.7821	Up (EB)	6.31	0
			Up (LB)	1.66	0.01
			Down (EB to LB)	-4.64	0
			Up (ES)	3.16	0.0002
			Down (ES to LS)	-2.14	0.003
Defensin 1	GAWM01019039	m.9997	Up (EB)	2.6	0.006
			Up (ES)	2.7	0.012
Defensin 2	GAWM01019040	m.9998	Up (LB)	2.72	0.012
			Up (ES)	3.87	0.002
Cecropin	GAWM01000005	m.10000	Up (EB)	4.57	8.12E-13
			Up (LB)	3.42	2.31E-08
			Down (EB to LB)	-1.14	0.009
			Up (ES)	3.11	6.15E-05
			Down (ES to LS)	-2.69	1.00E-04
**Regulators**					
PIRK/PIMS	GAWM01010231	m.4008	Down (EB)	-2.28	1.38E-05
			Down (LB)	-2.38	5.40E-10
Caspar, FAF1	GAWM01012793	m.49687	Down (EB to LB)	-0.94	1.00E-03
PGRP-SC2/SC3	GAWM01018647	m.9236	Up (LB)	3.96	3.00E-10
			Up (EB to LB)	2.27	8.00E-04

^a^EB, early blood fed; LB, late blood fed; ES, early sucrose fed; LS, late sucrose fed.

All five AMP target genes were differentially expressed following blood feeding (Table 4, Figure 2). Genes for the AMPs *attacin-like* and *attacin* were both highly upregulated after blood feeding, but differed in their temporal expression patterns, with *attacin-like* being late-induced, and *attacin* being early-induced (Table 4, Figure 2). Sucrose feeding also caused upregulation of each gene, with similar patterns in temporal expression. Both *defensin* paralogs were upregulated in midges in the early sucrose-fed transcriptomes, but they differed in their upregulation in response to blood feeding, with one being early-blood and one being late-blood induced (Table 4, Figure 2). Expression of *cecropin* was upregulated by both sucrose and blood feeding, with early responses being significantly higher than late (P ≤ 0.009; Table 4).

**Figure 3:**
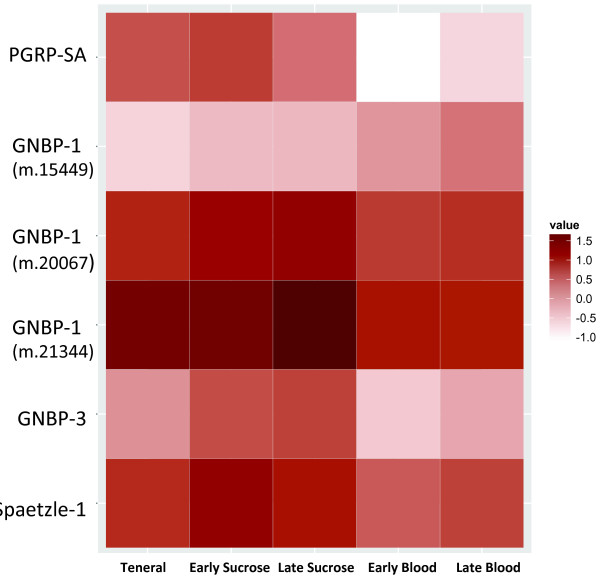
**Transcriptome-wide differential expression analyses of selected** ***Culicoides sonorensis*** **Toll genes.** Early transcriptomes are 2, 6, 12 h post ingestion (pooled) and late transcriptomes are 36 h post ingestion, for each diet (blood or sucrose). Teneral midges were newly emerged and unfed. Log_10_ FPKM values indicated in legend of the heat map. Further description of these genes can be found in Table 2, and fold-change values and statistics can be found in Table 5.

Many Toll pathway components were differentially expressed in midges following blood or sucrose feeding (Table 5). *PGRP-SA* was downregulated in both early and late blood-fed midges, but most of the other upstream signaling components were significantly upregulated (P ≤ 0.01) in either blood or sugar fed midges (except one of the GNBP1; Table 5, Figure 3). In contrast, *toll* receptors and *dorsal* transcription factors were downregulated after blood feeding (Table 5). The signaling components *myd88*, *pelle*, *tube* and *cactus* were all upregulated in early blood fed midges. Systemic responses to conditions in the gut suggest that there is communication between these two body compartments in midges. Further, the expression patterns of Toll components could be a glimpse into feedback mechanisms designed to quell the systemic response to proliferating gut microbiota which are immunostimulatory, yet are not invasive or threatening to the midge.

**Table 5:** **Toll pathway genes differentially expressed with diet**

Description	Acc. no.	Seq. no.	Diff. exp. ^a^	Log _2_fold chg	P-value
**Upstream signaling**					
PGRP-SA	GAWM01018051	m.7996	Down (EB)	-5.35	2.00E-04
			Down (LB)	-3.06	6.05E-06
GNBP1	GAWM01002165	m.15449	Up (LB)	3.7	1.34E-07
			Up (EB to LB)	3.25	1.00E-04
	GAWM01003712	m.20067	Up (LS)	1.15	3.18E-03
	GAWM01004143	m.21344	Down (EB)	-1.73	3.00E-04
			Up (LS)	0.78	1.00E-02
GNBP3	GAWM01011997	m.46772	Up (LB)	2.71	1.35E-06
			Up (EB to LB)	4.44	2.07E-07
			Up (LS)	2.01	2.00E-04
spaetzle1	GAWM01015015	m.58907	Up (LB)	2.6	1.78E-10
			Up (EB to LB)	3.55	9.54E-09
**Receptors**					
toll	GAWM01015594	m.61585	Down (LB)	-1.35	4.00E-03
	GAWM01019001	m.9915	Down (EB to LB)	-2.58	1.00E-04
	GAWM01013057	m.50841	Down (LB)	-1.5	1.00E-03
			Down (EB to LB)	-1.94	1.00E-04
	GAWM01013058	m.50847	Down (LB)	-2.56	3.00E-04
			Up (EB to LB)	1.95	7.00E-03
**Cell signaling**					
MYD88	GAWM01018790	m.948	Up (EB)	1.28	5.29E-06
			Down (EB to LB)	-0.92	4.00E-04
Pelle	GAWM01001221	m.12898	Up (EB)	1.44	2.00E-03
			Up (LB)	1.23	3.00E-03
Tube	GAWM01007838	m.32196	Up (EB)	2.37	2.60E-14
			Up (LB)	1.3	9.22E-05
			Down (EB to LB)	-1.07	1.00E-04
			Up (LB)	1.3	9.22E-05
Cactus	GAWM01009580	m.37494	Up (EB)	0.85	6.00E-03
			Down (EB to LB)	-0.89	4.00E-03
**Transcription**					
dorsal	GAWM01010293	m.40244	Down (LB)	-0.99	7.00E-03
			Down (EB to LB)	-1.11	6.00E-03
	GAWM01010294	m.40249	Down (LB)	-1.12	2.00E-03
			Down (EB to LB)	-1.06	1.00E-02
	GAWM01010297	m.40255	Down (LB)	-1.21	8.00E-03

^a^EB, early blood fed; LB, late blood fed; ES, early sucrose fed; LS, late sucrose fed.

The negative regulators of the JAK/STAT pathway, *SOCS* and *PIAS*, were upregulated in midges early after blood feeding (P ≤ 0.000009; Table 6). In addition, although *hop* was upregulated after early blood feeding, expression of *STAT* transcription factors was downregulated in early blood-fed midges. Except for the *dome* receptors, expression levels of all JAK/STAT components returned to baseline (teneral) levels at 36 h post ingestion (Table 6). The phenomenon of blood feeding alone suppressing the JAK/STAT pathway would play an important role in the infection success of arboviruses present in the blood meal, since the expression of some antiviral genes is regulated by STAT [8]. We are currently investigating whether this early downregulation occurs locally in gut epithelial cells, which serve as the midge’s primary line of defense against arboviruses.

**Table 6:** **JAK/STAT pathway genes differentially expressed with diet**

Description	Acc. no.	Seq. no.	Diff. Exp. ^a^	Log _2_fold chg	P-value
**Receptors**					
Dome	GAWM01016156	m.64065	Down (LB)	-1.23	2.00E-04
			Down (EB to LB)	-1.69	6.83E-08
			Down (LS)	-0.94	1.00E-03
**Cell signaling**					
Hop	GAWM01005626	m.25895	Up (EB)	1.15	6.00E-05
			Down (EB to LB)	-1.23	1.15E-05
**Transcription**					
STAT	GAWM01007780	m.32041	Down (EB)	-3.54	4.77E-13
			Up (EB to LB)	3.41	1.03E-12
	GAWM01011778	m.45760	Up (EB to LB)	1.21	5.00E-04
	GAWM01013279	m.51827	Down (EB to LB)	-0.68	8.00E-03
**Regulators**					
SOCS	GAWM01008657	m.34752	Up (EB)	1.26	8.98E-06
			Down (EB to LB)	-1.18	1.25E-05
PIAS, sumo ligase	GAWM01011450	m.44071	Up (EB)	2.01	1.03E-10
			Down (EB to LB)	-2.22	1.37E-12
	GAWM01011451	m.44074	Up (EB)	2.29	1.93E-11
			Down (EB to LB)	-2.41	1.18E-12

^a^EB, early blood fed; LB, late blood fed; ES, early sucrose fed; LS, late sucrose fed.

Expression of other systemic immune components also changed significantly after blood feeding (P ≤ 0.00001). This included *tep3* [GenBank: GAWM01016118], which was downregulated nearly 4-fold early after blood feeding but then returned to baseline expression levels 36 h after the blood meal, and two genes for prophenoloxidase (*ppo*). One *ppo* gene [GenBank: GAWM01010754] was upregulated nearly 9-fold in early blood fed midges and over 1000-fold in late blood fed midges. However, the other *C. sonorensis ppo* [GenBank: GAWM01015170] was downregulated over 16-fold in early blood-fed midges before returning to the baseline expression level at 36 h post-blood feeding.

### AMP expression in the gut of female *C. sonorensis*

**Figure 4:**
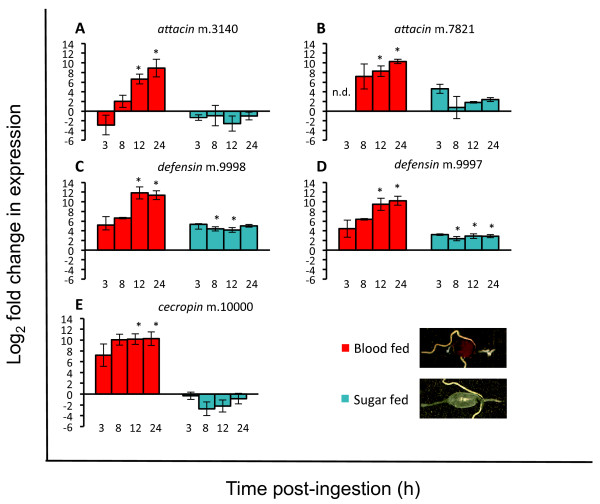
**qRT-PCR analysis of midgut antimicrobial peptide (AMP) expression in female** ***Culicoides sonorensis*** **.** Female midges were fed blood or sucrose and midguts (n = 15 per time-point) were dissected at 3, 8, 12, and 24 h after feeding. Relative AMP expression was determined using the methods of Pfaffl [31] for *attacin* m.3140 **(A)**, *attacin* m.7821 **(B)**, *defensin* m.9998 **(C)**, *defensin* m.9997 **(D)**, and *cecropin* m.10000 **(E)**, with teneral midges serving as the calibrator condition and incorporating the reference gene *EF1b.* Error bars represent SEM taken from three biological replicates. Asterisks denote change in expression from baseline (teneral) levels (P < 0.05). n.d., not determined (i.e., threshold cycle not crossed within 40 cycles). The details of each of the three biological replicates, including P-values, are available in Additional file 2.

As a follow up to the transcriptome-wide analysis of innate immune gene expression in female *C. sonorensis*, we performed tissue-specific qRTPCR analysis of AMP expression in the alimentary canal (“gut”). The aim was to more finely assess the temporo-spatial expression of these effector genes after blood and sucrose feeding. In congruence with our whole midge transcriptome-wide expression analyses (Table 4, Figure 4), blood feeding resulted in upregulation of all five AMPs in the gut (Figure 4). The *attacin-like* AMP was significantly upregulated in late blood-fed midges while *attacin* was upregulated early and sustained through 24 h post-blood ingestion (Figure 4A and B, respectively). On a whole-midge level, the two *defensin* genes showed different patterns of expression with *defensin* m.9997 being upregulated early after blood feeding, and *defensin* m.9998 being induced late after blood feeding (Table 4). However, in the gut-specific qRTPCR analysis, both *defensin* genes showed similar patterns of upregulation after blood feeding, and the fold-increase was significantly different from teneral midge expression levels at 12 and 24 h after blood feeding (Figure 4C and D). This suggests that the differential expression patterns seen in the transcriptome-level analyses would be attributable to tissues other than the alimentary canal, possibly the fat body. Both local (gut) and systemic (fat body) *defensin* responses to the ingested blood meal have been reported in other hematophagous arthropods [61–63]. Midge *cecropin* was upregulated at all four time points after blood ingestion, and expression levels were significantly different from teneral midges at 12 and 24 h post-blood feeding (Figure 4E). Sucrose feeding did not result in significant upregulation of *attacin-like*, *attacin* or *cecropin* in the alimentary canal, but did induce expression of both *defensin* genes. The expression of these AMPs after the blood meal is likely a consequence of altered microflora populations, whose proliferation would have an immunostimulatory effect on the gut epithelial cells. Intriguingly, this suggests that blood feeding alone indirectly impacts the conditions of the gut and, putatively, the vector competence of midges for pathogens in the blood meal.

## Conclusions

We demonstrated conservation of humoral immune components in the three major immune pathways of insects (Imd, Toll and JAK/STAT) in the *C. sonorensis* transcriptome. We have also provided insight into these defense pathways in the midge, by examining their patterns of expression on a transcriptome-wide level. We showed that blood feeding alone greatly impacts the expression of many components of these pathways, most importantly effector molecules such as AMPs, PPO and TEPs, which may be directly involved with the midge’s vector competence for pathogens. This knowledge allows us to take the next steps in assessing function by utilizing reverse-genetics (e.g. RNAi) approaches to more clearly define the role of the innate immune system in midge permissiveness or refractoriness for pathogens. Such studies will be aimed at revealing novel transmission-blocking and disease intervention strategies.

In this study, we did not explore the other arthropod immune and defense response components including the DUOX and JNK pathways, components of which have been found in our transcriptome but have not yet been completely characterized. These pathways as well as other defense systems such as iron sequestration, melanization and cellular responses will be an important focus of future studies aimed at fully characterizing the immune repertoire of this important vector species.

## Electronic supplementary material

Additional file 1: **Primer sequences used for qRT-PCR analyses of antimicrobial peptide gene expression in female** ***C. sonorensis*** **alimentary canal.** (PDF 50 KB)

Additional file 2: **Antimicrobial peptide (AMP) expression analysis using REST-MCS©.** Midges were fed blood or sucrose and processed as described in the text for qRTPCR of AMP gene expression, with three biological replicates (shown). A pairwise fixed allocation randomization test was performed using REST-MCS® to analyze AMP gene expression. P-values are for comparison to the calibrator state (teneral, unfed whole female midges) using the reference gene *EF1b*. Statistically significant P-values are shown in yellow. Red and blue represent upregulation and downregulation of target genes, respectively and grey indicates that threshold cycle was not crossed within 40 cycles (thus, no detectable expression). (PDF 50 KB)
