# Supplementary material for: Gene discovery and differential expression analysis of humoral immune response elements in female Culicoides sonorensis (Diptera: Ceratopogonidae)
Source: Parasit Vectors. 2014 Aug 21;7:388. doi: 10.1186/1756-3305-7-388 (PMC4158122; doi:10.1186/1756-3305-7-388)
Supplement: Supplementary file 1 — Additional file 1: Primer sequences used for qRT-PCR analyses of antimicrobial peptide gene expression in female C. sonorensis alimentary canal. (PDF 50 KB) [file 13071_2014_1568_MOESM1_ESM.pdf]

Additional file 1. Primer sequences used for qRT-PCR analyses of antimicrobial peptide gene expression in female *C. sonorensis* alimentary canal.

Gene	Unigene No.	Acc. No.	Primer Sequence
attacin-like	m.3140	GAWM01008443	Fwd: 5'-CATGGATTCAAGACCTCATCG-3'<br>Rev: 5'-CCAAGACCTACTCCAGTTGTA-3'
attacin	m.7821	GAWM01017969	Fwd: 5'-GGATTGTCGGGTAGTGTAAGT-3'<br>Rev: 5'-CCAAAGTGCTGTGTTGATCTC-3'
cecropin	m.10000	GAWM01000005	Fwd: 5'-AGCTCCTAGATGGAAAGGATG-3'<br>Rev: 5'-CTTTGTATCCTGCAACGACTG-3'
defensin	m.9997	GAWM01019039	Fwd: 5'-CAGACCAAATCCAAATCTCTCC-3'<br>Rev: 5'-GGCAGTATCCTGACTTGTACT-3'
defensin	m.9998	GAWM01019040	Fwd: 5'-TTCAACCAAGACTTTCATGCC-3'<br>Rev: 5'-CAAGTTCCATTACGACAATAGCC-3'
elongation factor 1b	m.31572	GAWM01010754	Fwd: 5'-ATCCGTGAAGAACGTCTCAAA-3'<br>Rev: 5'-CATGGCTTAACTTCGAGGATG-3'
