# Supplementary material for: Gene discovery and differential expression analysis of humoral immune response elements in female Culicoides sonorensis (Diptera: Ceratopogonidae)
Source: Parasit Vectors. 2014 Aug 21;7:388. doi: 10.1186/1756-3305-7-388 (PMC4158122; doi:10.1186/1756-3305-7-388)
Supplement: Supplementary file 2 — Additional file 2: Antimicrobial peptide (AMP) expression analysis using REST-MCS©. Midges were fed blood or sucrose and processed as described in the text for qRTPCR of AMP gene expression, with three biological replicates (shown). A pairwise fixed allocation randomization test was performed using REST-MCS® to analyze AMP gene expression. P-values are for comparison to the calibrator state (teneral, unfed whole female midges) using the reference gene EF1b. Statistically significant P-values are shown in yellow. Red and blue represent upregulation and downregulation of target genes, respectively and grey indicates that threshold cycle was not crossed within 40 cycles (thus, no detectable expression). (PDF 50 KB) [file 13071_2014_1568_MOESM2_ESM.pdf]

Additional file 2. Antimicrobial peptide expression analysis using REST-MCS©.

Blood

	Time-Point PI	Effector attacin-like m.3140	attacin m.7821	defensin m.9998	defensin m.9997	cecropin m.10000
Rep 1	3	0.938	0.093	0.001	0.882	0.04
	8	0.938	0.001	0.892	0.938	0.203
	12	0.001	0.001	0.001	0.001	0.001
	24	0.001	0.001	0.013	0.001	0.001
Rep 2	3	0.492		0.938	0.938	0.001
	8	0.947	0.89	0.458	0.016	0.001
	12	0.001	0.001	0.001	0.001	0.001
	24	0.001	0.001	0.001	0.04	0.001
Rep 3	3	0.492		0.938	0.888	0.938
	8	0.938	0.938	0.001	0.001	0.001
	12	0.001	0.001	0.001	0.001	0.001
	24	0.020	0.001	0.014	0.001	0.001

Sugar

	Time-Point PI	Effector attacin-like m.3140	attacin m.7821	defensin m.9998	defensin m.9997	cecropin m.10000
Rep 1	3	0.001	0.086	0.058	0.046	0.001
	8	0.044	0.248	0.001	0.001	0.042
	12	0.602	0.054	0.001	0.001	0.045
	24	0.847	0.112	0.06	0.06	0.001
Rep 2	3	0.046	0.001	0.001	0.001	0.051
	8	0.001	0.001	0.023	0.001	0.433
	12	0.4	0.097	0.001	0.001	0.2
	24	0.458	0.001	0.001	0.001	0.001
Rep 3	3	0.196	0.001	0.001	0.001	0.001
	8	0.001	0.099	0.042	0.042	0.001
	12	0.045	0.001	0.001	0.001	0.946
	24	0.151	0.001	0.001	0.001	0.452

yellow text = signficant change in expression

(red)	UP
(blue)	Down
(grey)	not detected
